# Chaotic Entanglement: Entropy and Geometry

**DOI:** 10.3390/e23101254

**Published:** 2021-09-26

**Authors:** Matthew A. Morena, Kevin M. Short

**Affiliations:** 1 Department of Mathematics, Christopher Newport University, Newport News, VA 23606, USA; 2 Integrated Applied Mathematics Program, Department of Mathematics and Statistics, University of New Hampshire, Durham, NH 03824, USA; Kevin.Short@unh.edu

**Keywords:** chaotic entanglement, chaotic systems, cupolets, entropy, unstable periodic orbits, geometry, lattices, coherent structures

## Abstract

In chaotic entanglement, pairs of interacting classically-chaotic systems are induced into a state of mutual stabilization that can be maintained without external controls and that exhibits several properties consistent with quantum entanglement. In such a state, the chaotic behavior of each system is stabilized onto one of the system’s many unstable periodic orbits (generally located densely on the associated attractor), and the ensuing periodicity of each system is sustained by the symbolic dynamics of its partner system, and vice versa. Notably, chaotic entanglement is an entropy-reversing event: the entropy of each member of an entangled pair decreases to zero when each system collapses onto a given period orbit. In this paper, we discuss the role that entropy plays in chaotic entanglement. We also describe the geometry that arises when pairs of entangled chaotic systems organize into coherent structures that range in complexity from simple tripartite lattices to more involved patterns. We conclude with a discussion of future research directions.

## 1. Introduction

In a series of recent papers, it has been shown that chaotic systems can be stabilized onto periodic orbits through the imposition of a control scheme that applies a set of impulsive kicks to the chaotic system along control planes or Poincaré sections that intersect the attractor of the system. The approach is an adaptation of a control scheme introduced by Hayes, Grebogi, and Ott [1]. However, the discovery of the stabilization property was a surprise given that in most instances, a given set of controls will lead to a unique periodic orbit, independently of the initial condition of the chaotic system. The resulting periodic orbits are called a cupolets (*C*haotic *U*nstable *P*eriodic *O*rbit-*LETS*) and are close approximations of the unstable periodic orbits (UPOs) that are generic for most chaotic systems. Similar properties have been found for a number of chaotic maps.

The robustness of the stabilization scheme is of great practical importance because it means that one can drive the chaotic system into a preselected state without any knowledge of the intial state of the chaotic system. The first application of this technique was the development of a secure chaotic communication scheme that passed only control information between the transmitter and receiver, so that the chaotic system in use was completely hidden from any eavesdropper. The robust stabilization property then allowed the transmitter and receiver to be initialized into the same state [2,3]. Further development along these lines led to the creation of a remote key generation scheme that addresses the problem of key distribution in standard secure communication approaches [4,5].

In a somewhat different guise, the cupolets themselves have proven useful when used as basis functions for approximations of many objects that have rich spectral content, including creation of an audio synthesizer [6], an audio compression method [7], and an image and video compression method [8]. Further, the rich spectral content of cupolets allowed for manipulation of the phases of the cupolet components to create an image compression method where the basis functions would span a continuum between a wavelet-like state and a sinusoidal-like state [9,10,11].

Subsequent work addressed the question of whether cupolets can be used to provide a “skeleton” for the chaotic system that enables targeting and steering of the chaotic system in some desired manner. By looking at the maps between Poincaré sections, it was possible to develop mapping functions from a set of bins defined on the Poincaré section(s) to the next intersection with the bins on a Poincaré section. This allowed methods of graph theory to be brought to bear to develop an algorithm to calculate an efficient, in some sense, low-energy transition from an initial trajectory to a target trajectory by riding along and transitioning between cupolets [12]. Further investigation revealed that certain cupolets could be defined as fundamental and others as composite, with the fundamental cupolets irreducible within a finite space of cupolets [13].

After this preliminary work, the question arose whether interacting chaotic systems could ever lock into mutually stabilizing periodic orbits, where the interaction would be mediated by a fixed exchange function. The basic question can be summarized under the assumption that the same impulsive control scheme is operating on two identical, chaotic systems (Systems I and II, say): when System I is stabilized onto a cupolet, if the output of System I is passed through an exchange function that converts the output into a control sequence that is imposed on System II, would System II then stabilize onto a cupolet and *are there ever cases where the output of System II, when passed through the same exchange function, would provide the control sequence that would keep System I stabilized onto its cupolet*? It was shown in [14] that such mutually stabilizing persistent states do indeed exist, and this type of interaction was termed *chaotic entanglement*. Further discussions of chaotic entanglement and comparisons with quantum entanglement are covered in [15]. This will be the jumping off point for the work in this paper, so more review is provided in Section 2 below.

Recent work has addressed the question of whether cupolets could be found in mathematical models of neurons, since one of the main exchange functions under consideration is an “integrate-and-fire” model taken from neuroscience research. In [16], it is shown that persistent mutual stabilization could be achieved between a pair of Fitzhugh Nagumo neurons (mathematical neuron models where each individual model is two-dimensional, but the combined system exhibits chaos). The mutual stabilization occurs when the exchange function exhibits properties related to “synaptic learning”, where the dynamics of the synapse change as a function of the passage of a neural firing. In this approach, the exchange function would change as signals passed through a synapse, so it does not utilize a fixed exchange function as in previous cupolet research; however, the synaptic learning setup does achieve the mutual stabilization using continuous dynamical equations that do not involve impulsive kicks. Subsequently, the neuron model research established that cupolets are indeed found in the three-dimensional Hindmarsh–Rose neuron model in the traditional sense, where pre-defined impulsive kicks are applied on Poincaré surfaces, leading to various forms of mutual stabilization [17].

We are aware that several studies, such as [18] and the aforementioned [16], have also examined interactions between chaotic systems that have led to periodic states, but to our knowledge none have reported results consistent with our formulation of chaotic entanglement. For instance, the procedure outlined in [18] describes synchronizing two coupled laser systems first into chaotic states and then into a quasi-periodic coupling, but this is all performed by carefully tuning the parameters to a desired set of values. Similarly, the more recent study discussed in [16] reports on driving two interacting, chaotic neuron models into mutual stabilization via an external signal that adjusts a parameter shared by the two neurons. Chaotic entanglement is distinguished from studies of this nature because it describes how two chaotic systems mutually stabilize one another onto cupolets, all while the system parameters remain fully in the chaotic regime. Once established, a chaotic entanglement and the stability of each cupolet are maintained intrinsically by each system’s dynamical behavior and will persist until the interaction is disturbed. The parameters of the systems are not changed in this process, and, in general it is very difficult even for one chaotic system to stabilize onto a periodic state, all from within a chaotic parameter regime.

In this paper, we address a number of new configurations that can be achieved in chaotic entanglement. Notably, we demonstrate a new framework in which the entanglement can be generated at multiple locations around the attractor of a given chaotic system. Whereas the previous framework restricted chaotic entanglement to occurring between two chaotic systems, the new configuration allows for a chaotic system to entangle with multiple other chaotic systems. This new multi-system entanglement allows for groups of entangled cupolets to be organized into various geometric structures that range from simple lattices to more involved patterns. We also characterize the entropy associated with chaotic entanglement according its Kolmogorov (or metric) entropy, which quantifies the rate in which dynamical systems generate information over time. Notably, chaotic entanglement is an entropy-reversing event, which is unusual in classical mechanics.

## 2. Background on Cupolets

Cupolets are a new class of waveforms that can be isolated from a given chaotic system through a control mechanism that stabilizes the normally-unstable periodic orbits of the chaotic system. The control mechanism applies small controls that prevent the chaotic dynamics from wandering off from a periodic orbit, and hence the cupolets are approximations of the unstable periodic orbits (UPOs) that are typically generic on chaotic attractors. Cupolets were originally detected when analyzing and experimenting with secure chaotic communication applications. The theory behind cupolets and their many application areas has appeared in numerous publications [2,9,10,12,14,19,20,21]. This section will provide a brief summary of the chaotic systems and control techniques that lead to cupolets, with more technical details available in [9,10,12,14,21].

### 2.1. Generating Cupolets

While cupolets can be stabilized from several chaotic systems, this paper will focus primarily on the double scroll attractor (also known as Chua’s attractor) [22]. Many variations of this basic system exist in the literature and it has been implemented in circuitry, so it is a useful and practical example of a system that can generate cupolets. In the equations below [22], the connection to circuit implementations lead to the association of the *v* variables with voltages, and the *i* variable with current:

(1a) $$\begin{array}{cc} {\dot{v}}_{C_{1}} & =\frac{G\left(v_{C_{2}}-v_{C_{1}}\right)-g\left(v_{C_{1}}\right)}{C_{1}},\\ {\dot{v}}_{C_{2}} & =\frac{G\left(v_{C_{1}}-v_{C_{2}}\right)+i_{L}}{C_{2}},\\ {\dot{i}}_{L} & =-\frac{v_{C_{2}}}{L}, \end{array}$$

where the non-linear resistance *g* is given by:

(1b) $$\begin{array}{cc} g(v) & =\left\{\begin{array}{cc} m_{1}v, & \mathrm{if}\;|v|\leq B_{p},\\ m_{0}\left(v+B_{p}\right)-m_{1}B_{p}, & \mathrm{if}\;v\leq -B_{p},\\ m_{0}\left(v-B_{p}\right)+m_{1}B_{p}, & \mathrm{if}\;v\geq B_{p}. \end{array}\right. \end{array}$$

**Figure 1:**
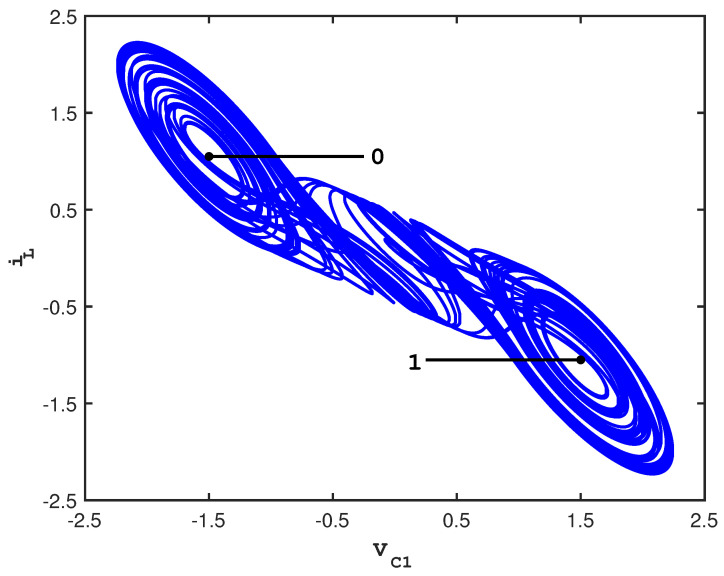
2D projection of the double scroll attractor showing the control surfaces [15].

For the usual parameters, $C_{1}=\frac{1}{9}$, $C_{2}=1$, $L=\frac{1}{7}$, G=0.7, $m_{0}=-0.5$, $m_{1}=-0.8$, and $B_{p}=1$, the double scroll exhibits chaotic behavior with an attractor that consists of two lobes, each of which surrounds an unstable fixed point [22]. Figure 1 shows a single, long chaotic trajectory tracing out the attractor.

In the figure, the cross-sections of two control planes are indicated by the straight lines; they emanate outward from the unstable fixed points that occur in the center of each lobe. The control scheme that is used to stabilize cupolets is adapted from one originally developed by Hayes, Grebogi, and Ott (HGO)for communication purposes [1,23]. In the HGO scheme, small perturbations are used to steer trajectories onto different lobes of the attractor, and by labeling the different lobes ‘0’ and ‘1’, it is possible to transmit a message. For cupolet generation, the use of adapted controls is described below, but the key idea is to examine how a chaotic system responds to the space of all possible periodic controls.

The procedure used for cupolet generation begins with setting up the two control planes on the attractor using a Poincaré surface of section and then partitioning each control plane into *N* small control bins, where typically N≈2000. In keeping with the original labeling of the lobes of the attractor in the HGO communication scheme, the control planes are assigned binary values ‘0’ and ‘1’ so that a binary symbolic sequence may be recorded whenever a trajectory intersects a given control plane. For later discussion, it is useful to give such a symbolic sequence a name, so it will be referred to as a *visitation sequence*. Except for when a trajectory passes through a Poincaré section (and hence, one of the control bins on the Poincaré section), the double scroll system evolves freely according to Equation (1). Perturbations are applied only when a trajectory hits a control bin. The option to perturb the trajectory is also encoded as binary; either give the system a kick to a nearby control bin (‘1’ control), or allow the trajectory to pass through the control bin (essentially) unperturbed (‘0’ control). We say “essentially” unperturbed, because every time the trajectory intersects a control plane and a ‘0’ control command is received, tiny *microcontrol* perturbations reset the trajectory to the center of the control bin through which it passes. In the cases where a ‘1’ control command is received, *macrocontrols* are applied that kick the trajectory to a nearby bin on the Poincaré section. Following the HGO approach, macrocontrol perturbations are defined to be the smallest perturbation along a control plane necessary to produce a change of lobe some *M*-many loops downstream in the visitation sequence. What this means in practice is that if a visitation sequence is *M*-bits long, a macrocontrol will shift the trajectory to a nearby control bin so that after *M* trips around the lobes of the attractor, the last bit of the visitation sequence will be different (and, of course, the trajectory will be on a different lobe from where it would be if left unperturbed). In this way, a chaotic system can be directed to follow any given visitation sequence by sending in an appropriately-defined string of control instructions, known as a *control sequence*. Since the number of control bins is typically large, the impulsive kicks along the control plane can be extremely small, yet when combined with the nature of chaotic dynamics, the downstream effects can be large and controllable.

**Figure 2:**
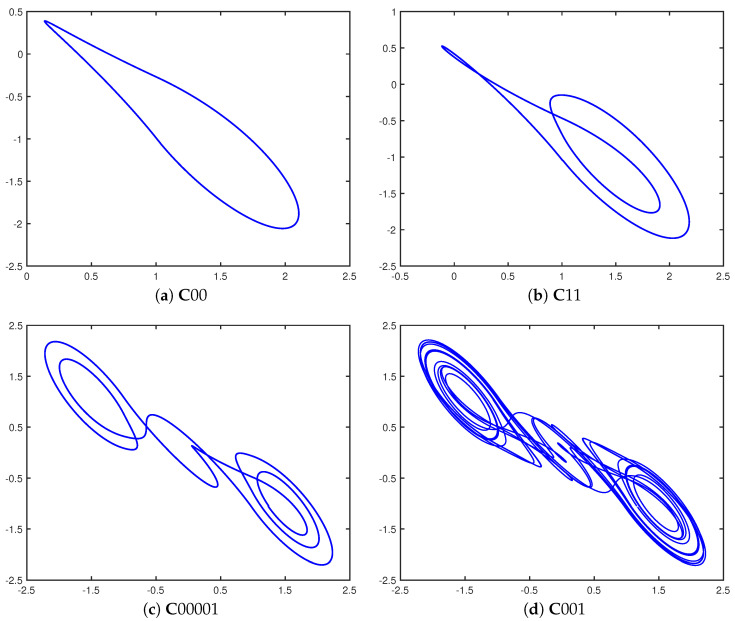
Cupolets of various periods belonging to the double scroll system [15]. The cupolets with the appended (repeated) control sequences are (**a**) C00, (**b**) C11, (**c**) C00001, and (**d**) C001.

Inspired by ideas from the study of impulsive differential equations [24] and specifically the reactions of dynamical systems to perturbations, Parker and Short [2] used the adapted control scheme to study the response of the double scroll system to the space of repeating binary control sequences. The unique, and somewhat surprising result, was that for almost all cases the double scroll system stabilizes onto a periodic orbit. In fact, the stabilization occurs regardless of the initial or current state of the system, or of the starting bit of each repeating control sequence. These periodic orbits have been given the name *cupolets* to indicate that they are *c*haotic *u*nstable *p*eriodic *o*rbit-*lets*. While this paper will deal only with the double scroll system, cupolets have also been found in chaotic maps and a variety of other continuous chaotic systems such as the Lorenz and Rössler systems. The examples of cupolets appearing in Figure 2 are generated by repetitively applying the indicated control sequences to the double scroll system. For simplicity, the cupolets are named with a leading “**C**” (for “control”) followed by their associated repeated control code. Later in this paper, visitation sequences associated with the given cupolets will be important, and those visitation sequences will have the letter “**V**” prepended to the binary visitation sequence—in some sense, a visitation sequence can be viewed as metadata associated with a cupolet.

### 2.2. Cupolet Properties and Stability

To summarize, as seen in Figure 2, cupolets are highly-accurate approximations to the UPOs of chaotic systems that are generated by the control scheme. Cupolets exhibit the properties of being stabilized independently of initial condition and also of being in one-to-one correspondence with the control sequences. The control bins can be made arbitrarily small so that the perturbations do not grossly alter the topology of the orbits on the chaotic attractor, and by increasing the *N*-bins and the *M*-downstream loops, the scale of the perturbations can be reduced accordingly. This suggests that cupolets are shadowing true periodic orbits, and theorems have been developed to establish conditions under which this holds [9,25,26,27,28]. What sets cupolets apart from UPOs is the ease with which cupolets are generated, since it is simple to run simulations where periodic control sequences are generated en masse, making the stabilization onto cupolets very rapid. Techniques from graph theory can be employed to speed up processing even further. Meanwhile, UPOs are typically stabilized via a combination of close-return algorithms and Newton interval operators. This process is cumbersome because it traditionally involves iteratively refining a brute-force search of the initial conditions of each periodic orbit. In contrast, the adapted HGO control scheme generates cupolets inexpensively and in large numbers; for example, over 8800 double scroll cupolets can be stabilized by implementing up to 16-bit control sequences.

To maintain stability of a cupolet, the only requirement is the repeated application of its control sequence to the system. If a different control were injected into the sequence, it would induce the system to destabilize from a stabilized cupolet and revert to chaotic behavior. If a second sequence of periodic controls were then applied, the chaotic system would eventually restabilize onto the cupolet associated with the new control sequence, after an intermediary transient phase as the system restabilizes. Any transient is the result of the trajectory evolving while the underlying chaotic system sifts through all possible states until it reaches one where the behavior of an UPO falls into synchrony with the control sequence, thus stabilizing the new cupolet. Cupolet restabilization is guaranteed because of the one-to-one relationship between cupolets and the binary control sequences. This has enabled an approach to navigating around an attractor by transitioning between cupolets or segments of cupolets, simply by switching control sequences. Details and efficient algorithms for planning the transition strategy appear in [12].

## 3. Chaotic Entanglement

In a recent series of papers [14,15,21], it is shown that pairs of chaotic systems that are capable of producing cupolets may be able to interact in a way that causes them to *chaotically entangle*, by which we mean that through their interaction they fall into a state of mutual stabilization onto their cupolets. For this to occur, the two chaotic systems are assumed to be connected via an *exchange function* that exchanges control information and, in a manner that will be detailed below, when one system is induced to collapse onto a cupolet state, that cupolet state passes into the interaction function which then induces the second chaotic system to collapse onto its own cupolet state. The mutually stabilizing feedback loop is then closed when, and if, the second cupolet state, when passed through the same interaction function, causes the first chaotic system to remain stabilized onto the first cupolet. This mutually stabilized, entangled state causes the cupolets to remain deterministically linked. The entanglement is persistent, but if one cupolet is disturbed from its periodic orbit, it subsequently affects the stability of the partner cupolet, and vice versa. This, and related properties evoke several connections to quantum entanglement, as discussed in [15]. In the cited references, hundreds of entangled cupolet pairs have been identified for the double scroll system, using several classes of exchange functions.

The production of chaotic entanglement is mediated by an interaction function we will call the exchange function, since it exchanges state information for control information and it will define the interaction between the two chaotic systems and their cupolets within the context of the control scheme described above. A variety of exchange functions have been studied, including integrate-and-fire functions that are used in laser or brain neuron research, moving average filters, and other filters similar to FIR filters. Exchange functions are described more fully in [14] and are considered to represent the environment or medium in which the interacting systems are found. Importantly, the same exchange function will be applied to both the forward interaction between the cupolets and the feedback interaction as well, since the exchange function is taken to be external to the chaotic dynamics. The input to the exchange function will be the visitation sequence of a given cupolet, and the output will be a set of control bits that are applied to the partner cupolet; chaotic entanglement occurs when the visitation sequences and the control sequences fall into serendipitous sympatico states. These varying exchange functions mentioned have all successfully induced chaotic entanglement in the double scroll system.

In Section 2, a cupolet’s visitation sequence was defined to be the binary sequence of lobes that its orbit visits. Consequently, visitation sequences define a type of symbolic dynamics for chaotic systems on the periodic orbits traced out by the cupolets. These symbolic sequences then become the inputs to the exchange functions and thus define the output controls that are applied to the second chaotic system to produce the partner cupolet. Chaotic entanglement results when the visitation sequences of the interacting cupolets turn into the control sequences necessary to keep the same cupolets stabilized.

We now provide a straightforward demonstration of chaotic entanglement using generic cupolets $C_{A}$ from System I and $C_{B}$ from System II, say. Beginning with System I having already been stabilized onto cupolet $C_{A}$, the visitation sequence of this cupolet is passed as input to an exchange function. The exchange function takes the visitation sequence and converts it to an output sequence of bits that is termed the *emitted sequence*. This emitted sequence is then turned into the control sequence that is applied to cupolet $C_{B}$ of System II. Since the interaction is a two-way interaction, the visitation sequence traced out by cupolet $C_{B}$ then passes through the same exchange function, and the resulting emitted sequence feeds back as control instructions to $C_{A}$. Thus, cupolets $C_{A}$ and $C_{B}$ are in a feedback loop, receiving and transmitting control information via the exchange function. The key concept is that there are cases where the emitted sequence from cupolet $C_{A}$ matches the control sequence needed to maintain the stability of cupolet $C_{B}$, while at the same time the emitted sequence of cupolet $C_{B}$ matches the control sequence needed to maintain the stability of cupolet $C_{A}$. When this occurs, the cupolets have become trapped in a mutually-stabilizing feedback loop which we have defined as chaotic entanglement. The external controls applied to cupolet $C_{A}$ can be removed once the mutual stabilization has occurred.

**Figure 3:**
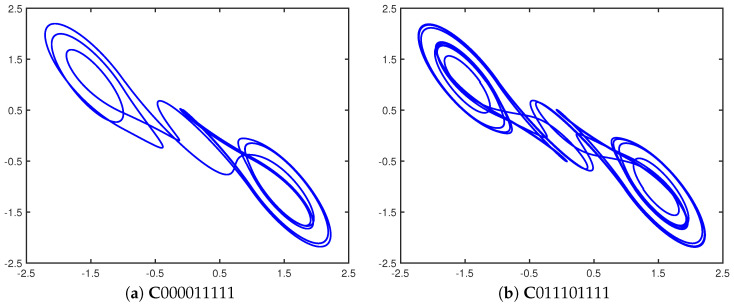
Entangled cupolets (**a**) C000011111 (period 9) and (**b**) C011101111 (period 18) [15]. The visitation sequences of these cupolets are V000111111 and V000000000111111111, respectively.

**Figure 4:**
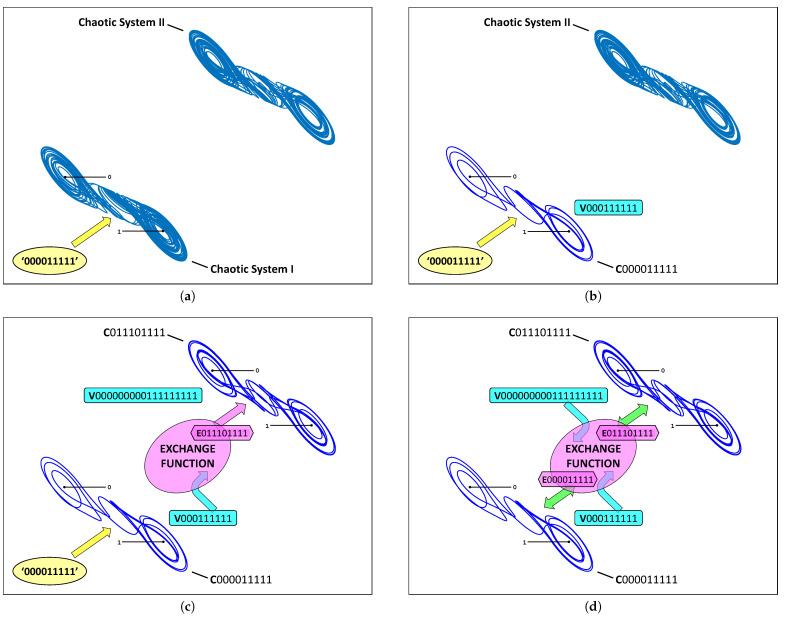
(Color online) Schematic illustration of chaotic entanglement as seen in [15]: in (**a**) a control sequence is applied to Chaotic System I via the (yellow) control pump. This causes System I to stabilize in (**b**) onto cupolet C000011111 with visitation sequence V000111111. In (**c**), a preponderance exchange function converts V000111111 into the emitted sequence, E011101111. When E011101111 is applied as a control code to Chaotic System II, the system stabilizes uniquely onto cupolet C011101111 with visitation sequence V000000000111111111. In (**d**), the exchange function converts V000000000111111111 into E000011111, which is then applied as control instructions to C000011111 of System I. The emitted sequence of each cupolet matches the control sequence of the opposite cupolet, and so the external control pumps seen in (**a**–**c**) are unnecessary and can be removed. Systems I and II are now chaotically entangled via their cupolets. This entanglement is summarized in Table 1. The orbits of these cupolets can be seen in more detail in Figure 3.

To make this visually evident, the following figures are reprinted from [15], with a summarized discussion. Consider the two cupolets shown in Figure 3, along with the cartoon depicted in Figure 4, which portrays the chaotic entanglement of cupolets C000011111 and C000011111 in a series of step-by-step illustrations. The process starts with two (uncontrolled) chaotic double scroll systems, Systems I and II. When the control sequence ‘000011111’ is applied to System I, it will stabilize onto cupolet C000011111. This step is illustrated in Figure 4a, where the (yellow) oval represents the external control pump. Cupolet C000011111 then produces the visitation sequence, V000111111, as shown in Figure 4b. This visitation sequence is then passed as input to the exchange function where it is operated on to produce a binary output that is transmitted to System II as an emitted sequence. The specific exchange function used here is a ‘preponderance’ exchange function (see [14]) that converts V000111111 into the emitted sequence E011101111 essentially by interpreting the bits of a visitation sequence as binary energy and imposing an energy threshold on the visitation sequence. The emitted sequence ‘011101111’ is applied as controls to System II, causing it to stabilize onto the cupolet C011101111 (as is evident since the emitted sequence actually *is* this second cupolet’s control sequence). This stage of the process is depicted in Figure 4c.

The feedback interaction now occurs in the reverse direction. The visitation sequence of cupolet C011101111 is V000000000111111111 (again see Figure 4c, which is transformed by the preponderance exchange function into the emitted sequence E000011111. Since this emitted sequence is the same as the external control sequence required to stabilize System I, when it is applied as a feedback control to System I, it maintains the stability of cupolet C000011111. Thus, the output of System I stabilizes System II, and the output of System II in turn stabilizes System I, and so we have established a mutually stabilizing, persistent feedback loop. The cupolets are now considered chaotically entangled, and their stabilities are guaranteed so long as their two-way interaction is undisturbed, as illustrated in Figure 4d. Table 1 summarizes the correspondence between the control, visitation, and emitted sequences of these cupolets.

The chaotic entanglement summarized in this section has been found with numerous forms of exchange functions and many different cupolet pairs, as shown in the references [14,15]. Another example has been found where the exchange function is the identity function, so the applied controls are determined purely by the lobe visitation sequence of the cupolets. This is known as *pure entanglement* since no intermediary exchange function is needed [21]. The use of cupolets has facilitated these explorations since they make the investigation more efficient, but these properties are expected to be shared by the true UPOs of the chaotic systems.

## 4. Entropy in Chaotic Entanglement

In recent years, entropy has emerged as a fundamental tool for characterizing the statistical uncertainty exhibited in both quantum and classical mechanics. In quantum mechanics, entropy is used to assess the strength of an entanglement [29]. In classical systems, entropy is inherently related to information theory as it is used to measure the rate in which a dynamical system generates information over time. Kolmogorov or metric entropy applies to classical dynamical systems and relies heavily upon this information-theoretic foundation [30,31,32]. Kolmogorov entropy essentially measures the rate of growth of sequences that a trajectory visits as it evolves on an the attractor.

To compute the Kolmogorov entropy of a dynamical system, begin with a sequence of samples of an associated trajectory made at regular time intervals Δt and consider a partition *B* of the phase space into hypercubes $\beta_{i}$. The ergodicity property of dynamical systems allows for time series averaging, with each sample representing the starting point of a sequence to produce the appopriate probability measures. Let $p\left(\beta_{i}\right)$ denote the probability that the dynamical system visits $\beta_{i}$ as it evolves through time. Then $p\left(\beta_{1},\beta_{2},\ldots ,\beta_{b}\right)$ gives the joint probability that the trajectory visits a sequence of hypercubes ${\left\{\beta_{i}\right\}}_{1}^{b}$. Here the enumeration of the β’s from 1 to *b* is merely a convenience meant to convey an arbitrary sequence of *b*-many visited hypercubes. The Kolmogorov entropy *K* is defined as the supremum over all partitions and all time intervals Δt as the sequence length *b* goes to infinity:

(2) $$K=\underset{B,\;\Delta t}{\sup}\;\left[\lim_{b\to \infty }\left(-\frac{1}{b\Delta t}\sum_{\beta_{1},\;\ldots ,\;\beta_{b}}p\left(\beta_{1},\ldots ,\beta_{b}\right)\ln p\left(\beta_{1},\ldots ,\beta_{b}\right)\right)\right],$$

where the sum is taken over all sequences of length *b* [33].

The rate in which information is generated by a classical system depends on the nature of the dynamics that it exhibits; for instance, whether the system is periodic, chaotic, or random. The Kolmogorov entropy can be used to distinguish between these different types of behaviors. For instance, Equation (2) gives K=0 for periodic or quasi-periodic systems, since once they have completed a full period, or traced out a torus, there is no further generation of new sequences. In contrast, K→∞ for random sequences since a random system will generate information at an unbounded rate and realize all possible sequences. In between are the chaotic trajectories for which 0<K<∞, meaning that chaotic systems generate sequences at finite rates [32,33]. This last result follows from the fact that although the dynamics of chaotic systems are deterministic, their aperiodicity and the geometry of their attractors are such that chaotic trajectories are confined to evolving along unique and non-stochastic paths on an attractor.

**Figure 5:**
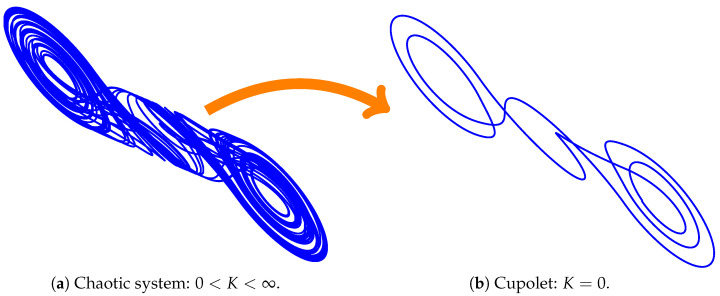
(Color online) This figure illustrates the entropy-reversing nature of cupolet stabilization, which can arise either via the control scheme described in Section 2 or via chaotic entanglement. In (**a**), we see an uncontrolled and freely-evolving chaotic system, which has positive and finite entropy. When it is stabilized onto the periodic orbit of a cupolet, seen in (**b**), the entropy decays to zero.

In chaotic systems, information is typically encoded in an associated symbolic dynamics, in which case entropy specifically measures the rate in which these systems generate new symbol sequences. The visitation sequences of the double scroll system provide such a symbolic dynamics, and hence entropy can be used to describe the dynamics before and after chaotic entanglement is established. As illustrated in Figure 5a, every freely-evolving chaotic system generates symbolic information at a positive and finite rate. As seen in Figure 5b, this entropy decays to zero when the chaotic system is directed onto a periodic or cupolet state, either by the control scheme described in Section 2, or when the system interacts with a second system and is induced into entanglement. Both scenarios involve the collapse of chaotic to periodic behavior, and the corresponding decrease to zero of each system’s entropy. In other words, chaotic stabilization and entanglement are each entropy-reversing events.

The discussion of chaotic entanglement in this paper has focused on induced entanglement, where an outside impetus is provided to drive System I onto a given cupolet. In that circumstance, one might point out that the reduction in entropy comes at the expense of input energy. However, as indicated in [14,15], the potential exists for serendipitous entanglement, since the two systems are fully in the chaotic regime of parameter space. This means that the dynamical evolution is aperiodic and, more importantly, the systems will visit the close vicinity of the unstable periodic orbits infinitely often. Further, since the interactions mediated by the exchange function are a property of the environment, not the chaotic systems, if one allows for the possibility that many thousands or millions or many moles of chaotic systems are present, then the opportunity for chaotic entanglement is significant, even without an external impetus to drive System I onto a cupolet. Thus, it may be possible for entropy reduction through chaotic entanglement to be a natural process.

## 5. Multi-System Entanglement

The framework described in Section 3 allows for the chaotic entanglement of two interacting chaotic systems. We now describe how this framework can be tailored to allow for the entanglement between more than two chaotic systems. In the new configuration, we imagine a degree of proximity between the lobes of an attractor, so that an individual lobe can individually send and receive control information. This allows each lobe to entangle with the lobes of other chaotic systems. The entanglement defined via this configuration is known as *multi-system entanglement* because it allows for chaotic systems with multi-lobed attractors to chaotically entangle with multiple systems. From here, lattices of entangled chaotic systems can be assembled, with chaotic entanglement playing the role of a bonding agent between each pair of interacting lobes.

### 5.1. Procedure

In this new framework, a local control code and local visitation sequence are associated to each lobe of the attractor. These are binary sequences that are similar to what will now be referred to as the global control code and global visitation sequence, respectively, which were discussed earlier in Section 3. A local control code contains the bits of the global control code that are applied exclusively along the control plane of an associated lobe. A local visitation sequence indicates the sequence of visits that the underlying cupolet or chaotic system makes to the associated lobe. Local control codes and local visitation sequences are effectively lobe-specific versions of their global namesakes in that they perform similar roles in the entanglement process, but do so in a way that allows entanglement to form at each lobe, rather than from the attractor as a whole.

To determine the local control code of a particular lobe, a cupolet’s global control code must first be repeated, if necessary, so that it has the same length as its global visitation sequence. This is natural to do because cupolets are generated in such a way that a visitation sequence either has the same length as one period of its cupolet’s control code, or is an integer multiple of that period. Next, the local control codes are initialized as initially-empty control arrays that have the same length as the global control code. Each local control code is then derived by sequentially comparing the bits of the global control code to the corresponding bits of the global visitation sequence. A ‘1’ in the visitation sequence, for instance, indicates that the cupolet is evolving around its 1-lobe, which means that the corresponding bit of the global control code would be applied along the control plane of the 1-lobe. This particular bit is copied from the global control code and inserted into the same location of the 1-lobe’s local control code. In this situation, the cupolet is not evolving around its 0-lobe, and so we simply insert a ‘0’ into the same location of the 0-lobe’s local control code. A zero that is inserted in this manner acts as a delay, or placeholder, until the cupolet next evolves around the lobe associated with local control code. This guarantees that the bits of a local control code are applied along the appropriate control plane in the order that they appear in the global control code.

Similarly, a ‘0’ in the global visitation sequence indicates that the cupolet is evolving around its 0-lobe, which means that the corresponding bit in the global control code would be applied along the control plane of the 0-lobe. This bit of the global control code is copied into the same location of the 0-lobe’s local control code. The ‘0’ in the visitation sequence indicates that the cupolet is not evolving around its 1-lobe, and so we insert a ‘0’ into the same location of the 1-lobe’s local control code to serve as a placeholder until the cupolet next evolves arounds its 1-lobe.

This process of distributing the bits of the global control code among the local control codes continues until the global control code and global visitation sequence have been completely compared bitwise. The resulting local control codes each have the same length as the global control code and contain the control instructions that are relevant to their particular lobe of the attractor. Were one to work backwards, a cupolet’s global control code can be recovered from its local control codes and its global visitation sequence. The local control codes thus collectively work in tandem to establish and maintain the stability of their associated cupolet, but separately they determine how the control scheme is implemented at each corresponding lobe. For the double scroll system, we obtain two local control codes, $C_{0}$ and $C_{1}$, that are associated with the 0- and 1-lobes, respectively.

Similarly, a local visitation sequence describes the sequence of visits that a trajectory makes to an associated lobe. Each time that a trajectory evolves around a particular lobe, the corresponding bit of the associated local visitation sequence is defined to be a ‘1’; otherwise, the corresponding bit is a ‘0’. For two-lobed attractors, such as the double scroll system, the local visitation sequence of the 1-lobe is the same as the global visitation sequence, while the local visitation sequence of the 0-lobe is the bitwise complement. Every local visitation sequence has the same length as the global visitation sequence. For the double scroll system, we obtain two such local visitation sequences, $V_{0}$ and $V_{1}$, for the 0- and 1-lobes, respectively.

As an example, the cupolet C0110111 has a global control code of length seven and a global visitation sequence, V11111110000111, of length 14. When we repeat the control code twice so that its bits align with those from the visitation sequence:

(3) C01101110110111.V11111110000111,

we see that the bits of the global control code associated with the 1-lobe control plane are ‘0110111’ and ‘111’, and that the 0-lobe control bits are ‘0110.’ Inserting the placeholder zeros in the correct locations gives $C_{0}=00000000110000$ and $C_{1}=01101110000111$. Regarding the cupolet’s local visitation sequences, observe that this cupolet traverses its 1-lobe seven times, which means that $V_{0}$ and $V_{1}$ begin as ‘0000000’ and ‘1111111’, respectively. C0110111 then makes four loops around its 0-lobe, followed by three loops about its 1-lobe, giving $V_{0}=00000001111000$ and $V_{1}=11111110000111$.

Each local visitation sequence can be converted by an exchange function into an emitted sequence that can then be applied as control information to the lobe of another chaotic system. This exchange of control information constitutes the interaction that is necessary for two chaotic systems to enter into entangled states according to the procedure described in Section 3, except that in the configuration outlined here, the interaction is carried out between the individual lobes of the two systems, rather than between the systems themselves. This variation of the chaotic entanglement process is known as multi-system entanglement because it allows for one chaotic system to entangle with multiple other systems, with each entanglement transpiring at one specific lobe of the attractor.

### 5.2. Demonstration

As a general demonstration, we consider two interacting chaotic systems, Systems A and B, as well as two cupolets, $C_{A}$ and $C_{B}$, that have already been stabilized from Systems A and B, respectively. Let $C_{0}^{A}$, $V_{0}^{A}$, and $E_{0}^{A}$ be the local control code, local visitation sequence, and local emitted sequence associated with the 0-lobe of cupolet $C_{A}$, and let $C_{1}^{B}$, $V_{1}^{B}$, and $E_{1}^{B}$ represent the same for the 1-lobe of cupolet $C_{B}$. We will use this example to demonstrate the entanglement between the 0-lobe of cupolet $C_{A}$ and the 1-lobe of cupolet $C_{B}$, and so neither the 1-lobe’s local control code of $C_{A}$, nor the 0-lobe’s local control code of $C_{B}$, will figure explicitly in our discussion.

As cupolet $C_{A}$ evolves around its attractor, its local visitation sequence $V_{0}^{A}$ is passed to an exchange function that performs a binary operation on $V_{0}^{A}$. The bits outputted from the exchange function form the emitted sequence, $E_{0}^{A}$, that is associated with the 0-lobe of $C_{A}$. $E_{0}^{A}$ is then transmitted as control instructions to the 1-lobe of System B. The interaction between Systems A and B now plays out in the reverse direction, from the 1-lobe of System B to the 0-lobe of System A. In particular, the emitted sequence $E_{0}^{A}$ is used to maintain the stabilization of System B onto cupolet $C_{B}$. As cupolet $C_{B}$ winds around its attractor, the bits of its local visitation sequence $V_{1}^{B}$ are generated and processed by the same exchange function that operates on $V_{0}^{A}$ coming from the 0-lobe of System A. This exchange function converts $V_{1}^{B}$ to $E_{1}^{B}$, which is then applied as a control sequence to the 0-lobe of System A.

In order for the 0-lobe of System A and the 1-lobe of System B to chaotically entangle, the control bits of $E_{0}^{A}$ must match those of $C_{1}^{B}$ and the control bits of $E_{1}^{B}$ must match those of $C_{0}^{A}$. If so, then the 0-lobe of System A is transmitting the exact control information to the 1-lobe of System B that is necessary for maintaining the stability of cupolet $C_{B}$. At the same time, the 1-lobe of System B is sending the exact control instructions to the 0-lobe of System A that are necessary for maintaining the stability of cupolet $C_{A}$. Once this particular interaction has been established, then the 0-lobe of System A and the 1-lobe of System B have entered into mutually-stabilizing states that require no external application of controls in order to be maintained.

Note that the two chaotic systems involved in this example still have lobes of their attractors that are as yet unused and that therefore provide opportunities for additional entanglement. Namely, the 1-lobe of $C_{A}$ is available to entangle with a cupolet from some other chaotic system, and similarly for the 0-lobe of $C_{B}$. This is why the entanglement established in this manner is known as multi-system entanglement. From here, lattices can be assembled between pairs of entangled chaotic systems, leading to the formation of coherent structures of wide ranging complexity.

The multi-system entanglement described here is just as sensitive to disturbance as the entanglement that we have previously described in Section 3. For instance, if emitted sequence $E_{0}^{A}$ does not match the control code $C_{1}^{B}$, say, then System B will not receive the necessary controls that ensure the stability of cupolet $C_{1}^{B}$. In this situation, System B will no longer be directed along the orbit of cupolet $C_{B}$ and will instead revert to evolving chaotically around its attractor. Doing so will generate a new local visitation sequence at the 1-lobe of System B and then a new emitted sequence that is different from $E_{1}^{B}$. This will ultimately induce a similar destabilization of System A from $C_{A}$.

### 5.3. Geometric Structures in Chaotic Entanglement

**Figure 6:**
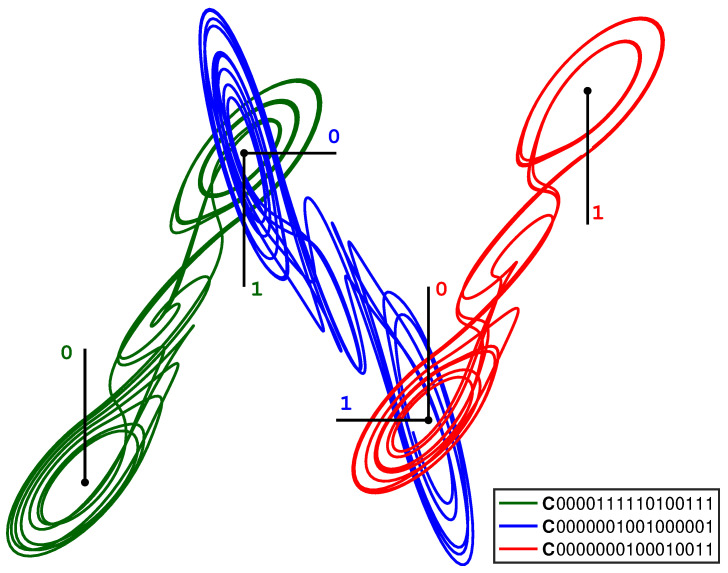
(Color online) A lattice of three double scroll systems that are chaotically-entangled via the cupolets C0000111110100111, C0000001001000001, and C0000000100010011. The 1-lobe of C0000111110100111 entangles with the 0-lobe of C0000001001000001, whose 1-lobe in turn entangles with the 0-lobe of C0000000100010011. An IntegrateAndFire exchange function is used to manage each interaction. This figure also shows the associated control plane labels. The global and local control codes and visitation sequences of these cupolets can be found in Table 1, which also contains the local emitted sequences of these cupolets.

Figure 6 shows an example of multi-system entanglement in which a geometric lattice has been constructed from three entangled cupolets. Here, the 1-lobe of C0000111110100111 (seen as the leftmost cupolet in the figure) entangles with the 0-lobe of C0000001001000001 (the central cupolet of the figure), whose 1-lobe of entangles with the 0-lobe of C0000000100010011 (the rightmost cupolet in the figure). The opposite lobes of these cupolets are aligned together in this figure, but this is purely for illustration purposes. All three cupolets are period-16. Each entanglement is managed by the IntegrateAndFire(5,8) exchange function, the details of which are described in [14]. Table 2 lists the relevant global and local control codes, visitation sequences, and emitted sequences of these cupolets. The process in which these pairs of cupolets entangle is outlined below.

As seen in Figure 6, the 1-lobe of $C_{A}=C0000111110100111$ entangles with the 0-lobe of $C_{B}=C0000001001000001$. This can be confirmed by checking Table 1, where we find that the emitted sequence produced by this exchange function for the 1-lobe of $C_{A}$ is $E_{1}^{A}=0000000000000001$. After a cyclic rotation of its control code bits, $E_{1}^{A}$ matches the local control code of the 0-lobe of $C_{B}$, which is $C_{0}^{B}=0000000001000000$. Similarly, the 0-lobe emitted sequence of cupolet $C_{B}$ is $E_{0}^{B}=0100000000000000$, which, after a cyclic rotation, matches the local control code of the 1-lobe of cupolet $C_{A}$, which is $C_{1}^{A}=0000000000000001$. As a result, the cupolets C0000111110100111 and C0000001001000001 entangle via this particular combination of lobes. A similar process leads to the entanglement between the 1-lobe of cupolet $C_{B}$ and the 0-lobe of cupolet $C_{C}=C0000000100010011$.

**Figure 7:**
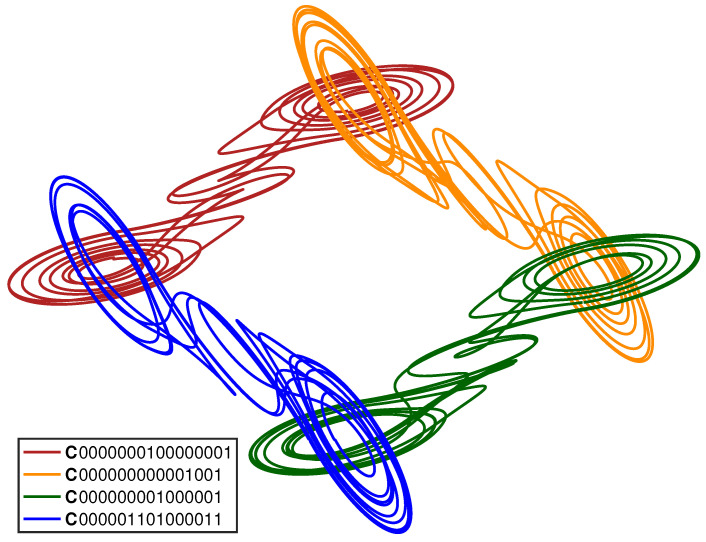
(Color online) A lattice of four, chaotically-entangled double scroll systems. The entangled cupolets are: C0000000100000001, C000000000001001, C000000001000001, C000001101000011, and C0000000000010001. An IntegrateAndFire exchange function is used to manage each entanglement.

**Figure 8:**
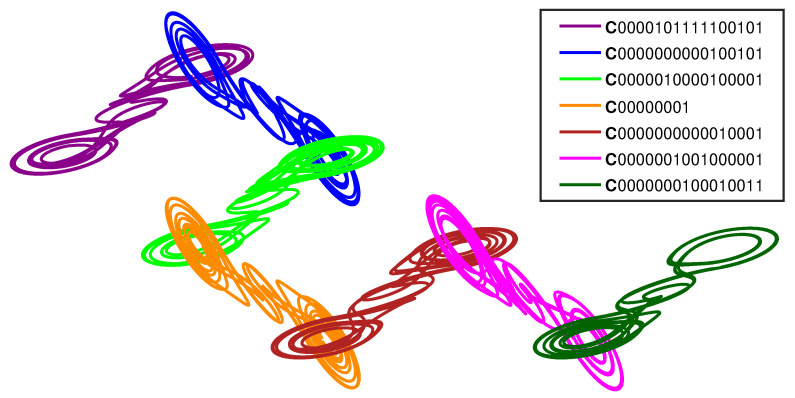
(Color online) A lattice of seven, chaotically-entangled double scroll systems. The entangled cupolets are: C0000101111100101, C0000000000100101, C0000010000100001, C00000001, C0000000000010001, C0000001001000001, and C0000000100010011. An IntegrateAndFire exchange function is used to manage each entanglement.

Figure 7 and Figure 8 show additional examples of the geometric patterns that can form from multi-system entanglement. Figure 7 depicts a loop of four entangled cupolets, while Figure 8 shows a chain of seven entangled cupolets. The coherent structures seen in these two figures are each generated using an IntegrateAndFire exchange function. As before, the opposite lobes of these cupolets are aligned together for illustration purposes.

Figure 7 is particularly interesting because it illustrates how a closed network of entangled cupolets may form. In particular, the 0-lobe of each cupolet entangles with the 1-lobe of its neighbor cupolet, and so on around the loop. This geometric structure is particularly sensitive to perturbation, given that the destabilization of just one of these four cupolets will evoke a chain reaction of destabilizations around the loop. Figure 8 is similarly intriguing because it demonstrates how large numbers of cupolets can be assembled into coherent structures via chaotic entanglement. This figure shows a chain of seven entangled cupolets, all of which are period-16.

## 6. Discussion and Future Research

Chaotic entanglement represents a recently-discovered phenomenon in which the dynamics of interacting chaotic systems are collapsed onto the orbits of cupolets, whose periodic behaviors are sustained so long as the interaction between the interacting systems is maintained. Previous research has documented numerous instances of chaotic entanglement in low-dimensional chaotic systems, but these results have so far been limited to two systems per entanglement. The new multi-system entanglement framework presented here removes the restriction of entangling with just one other system, so a given chaotic system can now entangle with multiple other chaotic systems through localized interactions at the invidivdual lobes of the associated attractors. From here, multi-entangled chaotic systems can be organized into various coherent structures that range in complexity from the simple tripartite lattice seen in Figure 6 to the closed loop structure of Figure 7 and the seven-cupolet lattice seen in Figure 8.

We stress that this application of chaotic entanglement remains at an information-theoretic level, and so additional work is necessary to relate this research directly to physical systems. In generating chaotic entanglement in an information-theoretic approach, one is free to define the interaction between entangled cupolets and hence how the control information that maintains their stability is exchanged. Even so, many of our exchange function are inspired by models of physical systems and anticipate methods that are easily implemented through linear shift registers or filters. For instance, the IntegrateAndFire exchange functions demonstrate how two chaotic systems representing neural or laser networks can interact.

As noted above, chaotic entanglement is an entropy-reversing event. This is not altogether surprising in the scenario where an external energy impulse is required to prime the generation of chaotic entanglement. However, as described above, it may naturally occur that a chaotic system tracks along an unstable periodic orbit and thus initiates an entanglement without any external energy. In whatever scenario, when the entanglement has been established, any external mechanism can be removed because at this point the entanglement can instead be maintained exclusively by the intrinsic dynamics of each (now-periodic) system. It is also worth emphasizing the sensitivity of chaotic entanglement to disturbance, as is discussed in [15]. Any disturbance to the stability of either cupolet of an entangled pair may be enough to compromise the entanglement, the effects of which would then transfer over to the partner cupolet by way of the exchange function. Both systems would then revert to chaotic behavior, causing their entropies to increase from zero to a positive value. This transition would not be instantaneous, though, because the previously-entangled systems would continue to evolve in close proximity of their respective cupolets for a period of time proportional to the local Lyapunov exponents of these cupolets. If their interaction is restored quickly enough, then the two systems would not have drifted too far from their previously-stabilized periodic orbits and could redirect each other back onto their respective cupolets, thus resuming their entanglement and a corresponding decay to zero in the overall entropy. In short, chaotic entanglement is an entropy-reversing event, but a fluid one at that, able to range from zero to positive and back.

**Figure 9:**
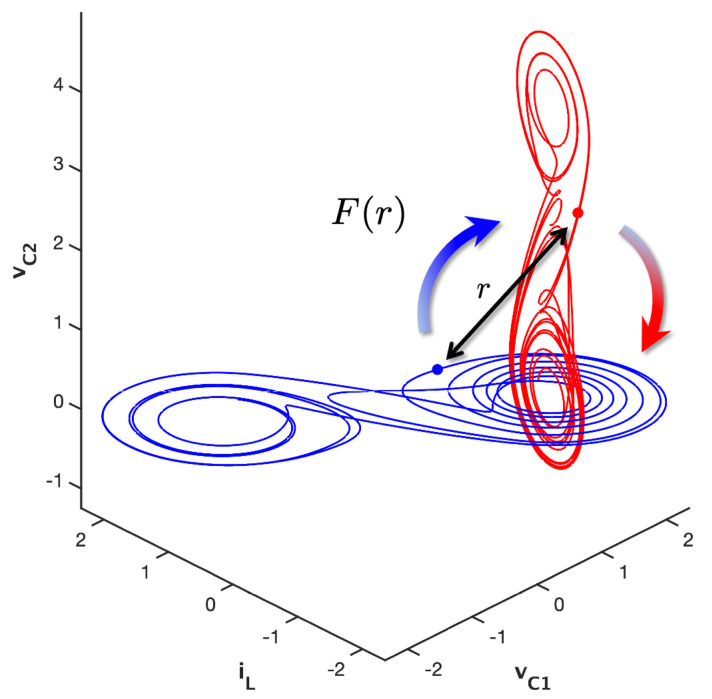
This figure illustrates how the interaction between two chaotic double scrolls would be defined via a short-range force, F(r). The strength of *F* is inversely related to the spatial separation, *r*, between the states of the two systems (at the same time). The idea is that *F* behaves as $1/r^{k}$ for some exponent k>0, so that F(r) is large when *r* is small, and vice versa. In this setup, *F* would play the same role as the controls that are implemented by the cupolet-generating control scheme described in Section 2. Under the right conditions, the continued interaction between the two evolving systems might lead them to stabilizing each other onto cupolets and then into chaotic entanglement.

Therefore, the next stage of research will be to identify physical interactions where induced chaotic entanglement can be found, which would demonstrate new interplay between chaotic and physical systems. One promising area where this may occur is in chaotic neuron entanglement, where the neuronal signals are naturally impulsive in a manner that is similar to how controls are implemented during the cupolet control scheme. Accordingly, we are also investigating certain Hamiltonian systems that are known to be chaotic, as well as several physical systems where an interaction is defined through a short-range force. Figure 9 illustrates this general idea, where we see two double scrolls systems interacting via a force, F(r), whose strength is inversely dependent on the separation distance, *r*, between the states of the two systems. The idea is that F(r) will be large when the states of the chaotic sytems are spatially close to each other, and small when the states of the systems are distant. In other words, *F* will behave as $1/r^{k}$, for some exponent k>0, which is consistent with the interactions that are defined via physical force laws like the well-known gravitational and Coulomb forces, which behave as $1/r^{2}$, or van der Waals forces, which can behave as $1/r^{6}$ [34].

In this setup, F(r) would assume the role of the controls that are implemented by the cupolet-generating control scheme described in Section 2. Rather than inducing perturbative “kicks” at select locations along a control plane, the effects of F(r) would be continuously applied to each chaotic system as it evolves around its attractor. This could lead to the stablization of each chaotic system onto a periodic orbit and the subsequent entanglement of the two systems.

Science is full of systems that interact and then remain in interaction. Covalent bonding in water molecules and the interactions caused by van der Waals forces are just two examples. If a van der Waals force law can be used as the interaction that induces two chaotic systems to entangle, then our work could be experimentally verifiable. If we can identify several physical interactions that lead to simulated chaotic entanglement, then the hope is that this research could lead to the discovery of chaotic entanglement in physical systems.

## Figures and Tables

**Table 1:** Summary of the chaotic entanglement seen in Figure 4. Cupolets C000011111 (of Chaotic System I) and C011101111 (of Chaotic System II) entangle because the control sequence required to sustain the stability of cupolet C011101111 is contributed by cupolet C000011111 via its emitted sequence, E011101111. In this case, a preponderance exchange function converts the visitation sequence into the emitted sequence. Similarly, the stability of C000011111 is maintained by the emitted sequence E000011111, which is generated by C011101111 via the same exchange function.

	Cupolet	Visitation Sequence	Emitted Sequence
Chaotic System I	C000011111	V000111111	E011101111
Chaotic System II	C011101111	V000000000111111111	E000011111

**Table 2:** This table contains the control code, visitation sequence, and emitted sequence information for the cupolets that are seen multi-entangled in Figure 6, which are cupolets $C_{A}=C0000111110100111$, $C_{B}=C0000001001000001$, and $C_{C}=C0000000100010011$. Specifically, this table lists these cupolets’ global control codes ($C_{A}$, $C_{B}$, and $C_{C}$, respectively), global visitation sequences ($V_{A}$, $V_{B}$, and $V_{C}$), local control codes ($C_{0}^{A}$, $C_{1}^{A}$, $C_{0}^{B}$, $C_{1}^{B}$, $C_{0}^{C}$, and $C_{1}^{C}$), local visitation sequences ($V_{0}^{A}$, $V_{1}^{A}$, $V_{0}^{B}$, $V_{1}^{B}$, $V_{0}^{C}$, and $V_{1}^{C}$), and local emitted sequences ($E_{0}^{A}$, $E_{1}^{A}$, $E_{0}^{B}$, $E_{1}^{B}$, $E_{0}^{C}$, and $E_{1}^{C}$). Each local emitted sequence is generated via the same IntegrateAndFire exchange function.

Cupolets		
C0000111110100111	C0000001001000001	C0000000100010011
$C_{A}=0000111110100111$	$C_{B}=0000001001000001$	$C_{C}=0000000100010011$
$V_{A}=1111000000000001$	$V_{B}=1000111000011111$	$V_{C}=0001100110000011$
$C_{0}^{A}=0000111110100110$	$C_{0}^{B}=0000001000000001$	$C_{0}^{C}=0000000000010000$
$C_{1}^{A}=0000000000000001$	$C_{1}^{B}=0000001000000001$	$C_{1}^{C}=0000000100000011$
$V_{0}^{A}=0000111111111110$	$V_{0}^{B}=0111000111100000$	$V_{0}^{C}=1110011001111100$
$V_{1}^{A}=1111000000000001$	$V_{1}^{B}=1000111000011111$	$V_{1}^{C}=0001100110000011$
$E_{0}^{A}=0100001000000000$	$E_{0}^{B}=0100000000000000$	$E_{0}^{C}=0000001000000001$
$E_{1}^{A}=0000000010000000$	$E_{1}^{B}=0000000000000001$	$E_{1}^{C}=0000000000000000$

## Data Availability

The data that support the findings of this study are available from the corresponding author upon reasonable request.
